# Climate-Related Local Extinctions Are Already Widespread among Plant and Animal Species

**DOI:** 10.1371/journal.pbio.2001104

**Published:** 2016-12-08

**Authors:** John J. Wiens

**Affiliations:** Department of Ecology and Evolutionary Biology, University of Arizona, Tucson, Arizona, United States of America; University of California-Berkeley, United States of America

## Abstract

Current climate change may be a major threat to global biodiversity, but the extent of species loss will depend on the details of how species respond to changing climates. For example, if most species can undergo rapid change in their climatic niches, then extinctions may be limited. Numerous studies have now documented shifts in the geographic ranges of species that were inferred to be related to climate change, especially shifts towards higher mean elevations and latitudes. Many of these studies contain valuable data on extinctions of local populations that have not yet been thoroughly explored. Specifically, overall range shifts can include range contractions at the “warm edges” of species’ ranges (i.e., lower latitudes and elevations), contractions which occur through local extinctions. Here, data on climate-related range shifts were used to test the frequency of local extinctions related to recent climate change. The results show that climate-related local extinctions have already occurred in hundreds of species, including 47% of the 976 species surveyed. This frequency of local extinctions was broadly similar across climatic zones, clades, and habitats but was significantly higher in tropical species than in temperate species (55% versus 39%), in animals than in plants (50% versus 39%), and in freshwater habitats relative to terrestrial and marine habitats (74% versus 46% versus 51%). Overall, these results suggest that local extinctions related to climate change are already widespread, even though levels of climate change so far are modest relative to those predicted in the next 100 years. These extinctions will presumably become much more prevalent as global warming increases further by roughly 2-fold to 5-fold over the coming decades.

## Author Summary

Climate change is an important threat to the world’s plant and animal species, including species on which humans depend. However, predicting how species will respond to future climate change is very difficult. In this study, I analyze the extinctions caused by the climate change that has already occurred. Numerous studies find that species are shifting their geographic ranges in response to climate change, typically moving to higher elevations and latitudes. These studies also contain valuable data on local extinctions, as they document the loss of populations at the “warm edge” of species’ ranges (lower elevations and latitudes). Here, I use these data to show that recent local extinctions related to climate change have already occurred in hundreds of species around the world. Specifically, among 976 species surveyed, local extinctions occurred in 47%. These extinctions are common across climatic zones, habitats, and groups of organisms but are especially common in tropical regions (which contain most of Earth’s species), in animals (relative to plants), and in freshwater habitats. In summary, this study reveals local extinctions in hundreds of species related to the limited global warming that has already occurred. These extinctions will almost certainly increase as global climate continues to warm in the coming decades.

## Introduction

Anthropogenic climate change may be a major driver of biodiversity loss in the next 100 years, but the possible impacts of climate change on species survival remain highly uncertain [1–3]. Global mean annual temperatures increased by ~0.85°C between 1880 and 2012 and are likely to rise by an additional 1°C to 4°C by 2100 [4]. Modeling studies have predicted that various levels of species loss will result from this future climate change, ranging from 0% to >50% of all species currently known [3]. This uncertainty has many sources (e.g., different climate models and different hypotheses about species dispersal). One of the most important sources of uncertainty hinges on the details of how species respond to climate change. For example, if species can evolve rapidly enough in response to changing climate, then species extinctions due to climate change might actually be limited [5,6].

Species can potentially respond to climate change in several ways. The most important case to consider may be that when the species’ present-day (realized) climatic niche no longer occurs within the species’ current geographic range (because of the potential for global extinction of the species under these conditions). In this case, the possible responses of the species include the following: (i) undergoing niche shifts, such that the species’ realized niche changes to incorporate these new climatic conditions (e.g., through plastic changes and/or by evolutionary adaptation to the modified abiotic and/or biotic conditions), (ii) dispersing to track the original climatic conditions over space (e.g., moving to higher latitudes or elevations), and (iii) going extinct [5–8]. While each of these responses has been shown in some cases (at least in local populations), the relative frequency of each is still unclear [7,8]. However, changes in species’ geographic ranges have been especially well documented [9–11].

**Fig 1:**
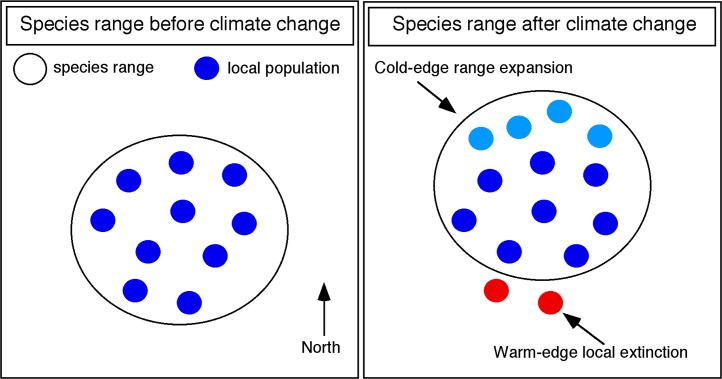
Hypothetical example illustrating the two components of a geographic range shift associated with climate change. The large open circle indicates the species’ overall geographic range. Small dark blue circles indicate populations before climate change. After climate change, the overall geographic range is shifted northward (large open circle), both through the range expansion (new populations; small light blue circles) added at the northern, “cold” edge of the species range and range contraction (local extinction of original populations; small red circles) at the southern, “warm” edge of the species range. Similar patterns occur for range shifts along an elevational gradient. Modified from Cahill et al. [12].

These data on geographic range shifts contain important but underutilized information on how species respond to climate change. Range shifts observed under climate change typically involve an overall shift towards higher latitudes and higher elevations [9–11]. These shifts can be composed of one (or both) of two types of changes (Fig 1): (i) range expansions at the cool edge of the species range (higher latitudes and elevations) and (ii) range contractions at the warm edge (lower latitudes and elevations). The presence of warm-edge contractions is critically important. A warm-edge contraction occurs when populations from one or more localities at the lowest latitudes or elevations of a species’ regional distribution disappear (i.e., are inferred to no longer occur at those localities), leading to an overall shift in the species range towards higher latitudes or elevations. These contractions indicate that species are failing to shift their niches sufficiently to tolerate these new conditions and that these populations are instead going extinct (referred to as “local extinction” hereafter). This must be true regardless of the specific mechanism of local extinction (e.g., elevated death rates, increased emigration, or declining recruitment). The many papers that have assessed range shifts and that have included surveys of warm-edge populations can therefore provide a wealth of data about which species have (and have not) undergone local extinctions potentially related to climate change. These data are particularly useful because published papers on range shifts need not be strongly biased towards documenting warm-edge contractions, given that many studies that included data on warm edges also surveyed the cool edge. Thus, even though studies that failed to find any range shifts might go unpublished (a potential source of bias), studies that documented an overall range shift need not show a warm-edge contraction.

Here, I analyze the extensive data on range shifts to examine the prevalence of local extinctions related to modern climate change. I also provide a synthesis of inferred local extinction across habitats, climatic zones, and taxonomic groups. I systematically searched the literature for studies that examined shifts in species’ ranges at their warm edges, shifts that were considered (in the original studies) to be related to current climate change. Hundreds of examples of local extinctions were found across diverse climatic zones, habitats, and taxonomic groups. Not all species exhibiting range shifts showed warm-edge contractions, but ~50% of the species surveyed had local extinctions inferred to be related to climate change. These results suggest that even the relatively small changes in climate that have already occurred are sufficient to cause widespread local extinctions and that many species may be unable respond to climate change fast enough to avoid extinction as global climate warms even further.

## Results

The Web of Science was searched repeatedly between December 2014 and March 2016 using keywords related to climate change, range shifts, and local extinctions (see Materials and Methods). All studies that monitored the warm edge of at least one species’ range and that tied their results to climate change with explicit statistical analyses were included. Importantly, studies can document overall range shifts but need not find that the warm-edge populations that they examined had local extinctions.

A total of 27 studies (Table 1; [13–39]) met all the necessary criteria to address potential climate-associated warm-edge range shifts (see Materials and Methods). The sampled species were broadly distributed across clades (e.g., animals = 716; plants = 260) and regions (e.g., Asia = 332; Europe = 268; Madagascar = 30; Oceania = 58; North America = 233; South America = 55). Among the 976 unique species surveyed, 460 species had warm-edge contractions, and 516 did not (S1 Appendix). Therefore, local extinctions related to climate change are already very common (47.1% of species examined), even given the relatively modest rise in global temperatures that has occurred so far (less than 1°C increase in global mean annual temperature; [4]).

**Table 1:** Summary information on the 27 range-shift studies used to document local extinctions related to climate change. Studies are listed alphabetically by first author. The major taxonomic group surveyed is given (Taxon, all groups are animals except for “Plant”), along with the total number of species surveyed (Total Species), the percentage of those species with one or more local extinctions (% Local Extinction), the general habitat type (Habitat; including terrestrial, freshwater, and marine), the climatic region (tropical-subtropical versus temperate), the geographic region where the study was conducted (note that North America here extends to Central America), the type of range shift (latitudinal, elevational), the dates of the initial survey and the resurvey, and the duration in between (for surveys and/or resurveys spanning multiple years, the midpoint of each was used to calculate the duration).

Reference	Taxon	Total Species	% Local Extinction	Habitat	Climatic Region	Geographic Region	Range Shift	Initial Survey	Resurvey Date	Duration
Angelo and Daehler [13]	Plant	4	50	Terrestrial	Tropical	Oceania (Hawaii)	Elevational	1966–1967	2008	41.5
Beever et al. [14]	Mammal	1	100	Terrestrial	Temperate	North America	Elevational	1898–1956	2003–2006	77.5
Brusca et al. [15]	Plant	27	56	Terrestrial	Tropical	North America	Elevational	1963	2011	48
Chen et al. [16]	Insect	208	56	Terrestrial	Tropical	Asia	Elevational	1965	2007	42
Comte and Grenouillet [17]	Fish	31	74	Fresh.	Temperate	Europe	Elevational	1980–1992	2003–2009	20
Dieker et al. [18]	Insect	2	50	Terrestrial	Temperate	Europe	Elevational	1958–1986	2008–2009	36.5
Felde et al. [19]	Plant	105	9	Terrestrial	Temperate	Europe	Elevational	1900	2008	108
Forero-Medina et al. [20]	Bird	55	29	Terrestrial	Tropical	South America	Elevational	1969	2010	41
Franco et al. [21]	Insect	3	100	Terrestrial	Temperate	Europe	Latitudinal	1970–1999	2004–2005	20
Freeman and Freeman [22]	Bird	54	74	Terrestrial	Tropical	Oceania (New Guinea)	Elevational	1965	2012	47
Hiddick et al. [23]	Marine invertebrates	65	55	Marine	Temperate	Europe	Latitudinal	1986	2000	14
Hitch and Leberg [24]	Bird	1	100	Terrestrial	Temperate	North America	Latitudinal	1967–1971	1998–2002	31
Menendez et al. [25]	Insect	39	54	Terrestrial	Temperate	Europe	Elevational	1981–1993	2006–2007	24
Moritz et al. [26]	Mammal	27	41	Terrestrial	Temperate	North America	Elevational	1914–1920	2003–2006	87.5
Myers et al. [27]	Mammal	8	12	Terrestrial	Temperate	North America	Latitudinal	1883–1980	1981–2006	62
Nye et al. [28]	Fish	28	50	Marine	Temperate	North America	Latitudinal	1968	2008	40
Perry et al. [29]	Fish	10	40	Marine	Temperate	North America	Latitudinal	1997	2001	24
Ploquin et al. [30]	Insect	16	69	Terrestrial	Temperate	Europe	Elevational	1988–1989	2007–2009	19.5
Pomara et al. [31]	Squamate	1	100	Terrestrial	Temperate	North America	Elevational	1965	2008	43
Raxworthy et al. [32]	Amphibian-Squamate	30	37	Terrestrial	Tropical	Madagascar	Elevational	1993	2003	10
Rowe et al. [33]	Mammal	4	25	Terrestrial	Temperate	North America	Elevational	1927–1929	2006–2008	79
Rubal et al. [34]	Mollusca	7	29	Marine	Temperate	Europe	Latitudinal	1917, 1940	2011	94
Sheldon [35]	Insect	1	0	Terrestrial	Temperate	North America	Elevational	1977–1978	2006	28.5
Telwala et al. [36]	Plant	124	60	Terrestrial	Tropical	Asia	Elevational	1849–1850	2007–2010	159
Tingley et al. [37]	Bird	92	25	Terrestrial	Temperate	North America	Elevational	1900–1930	1980–2006	78
Warren and Chick [38]	Insect	2	0	Terrestrial	Tropical	North America	Elevational	1973–1974	2012	38.5
Zuckerberg et al. [39]	Bird	31	71	Terrestrial	Temperate	North America	Both	1980–1985	2000–2005	20

These 976 species spanned many clades, habitats, and regions (Table 1; S1 Appendix). Comparison between those species that showed warm-edge contractions and those that did not provides potential insights into which species may be most sensitive to climate change, in terms of the climatic zones and habitats that they occur in and the clades that they belong to. Furthermore, there is no evidence that there were more species with local extinctions in studies that ended more recently, were of longer duration, or began earlier (based on midpoints for ranges of values; Table 1). Specifically, regression analyses of the proportion of species with local extinctions against (i) the study end date, (ii) the duration of the study, and (iii) the study start date all yielded nonsignificant results (end date: *r*^2^ = 0.001, *p* = 0.8910; duration: *r*^2^ = 0.045, *p* = 0.2896; start date *r*^2^ = 0.047, *p* = 0.2788; after removing nine studies with four or fewer species: end date: *r*^2^ = 0.146, *p* = 0.1181; duration: *r*^2^ = 0.132, *p* = 0.1376, but unexpectedly trending towards fewer extinctions in studies with longer durations; start date *r*^2^ = 0.177, *p* = 0.0821, with more extinctions in studies beginning more recently, not earlier). Therefore, the frequency of local extinctions was initially compared across species in different studies, regardless of differences in the duration, beginning, or end date of the study in which they were surveyed.

**Fig 2:**
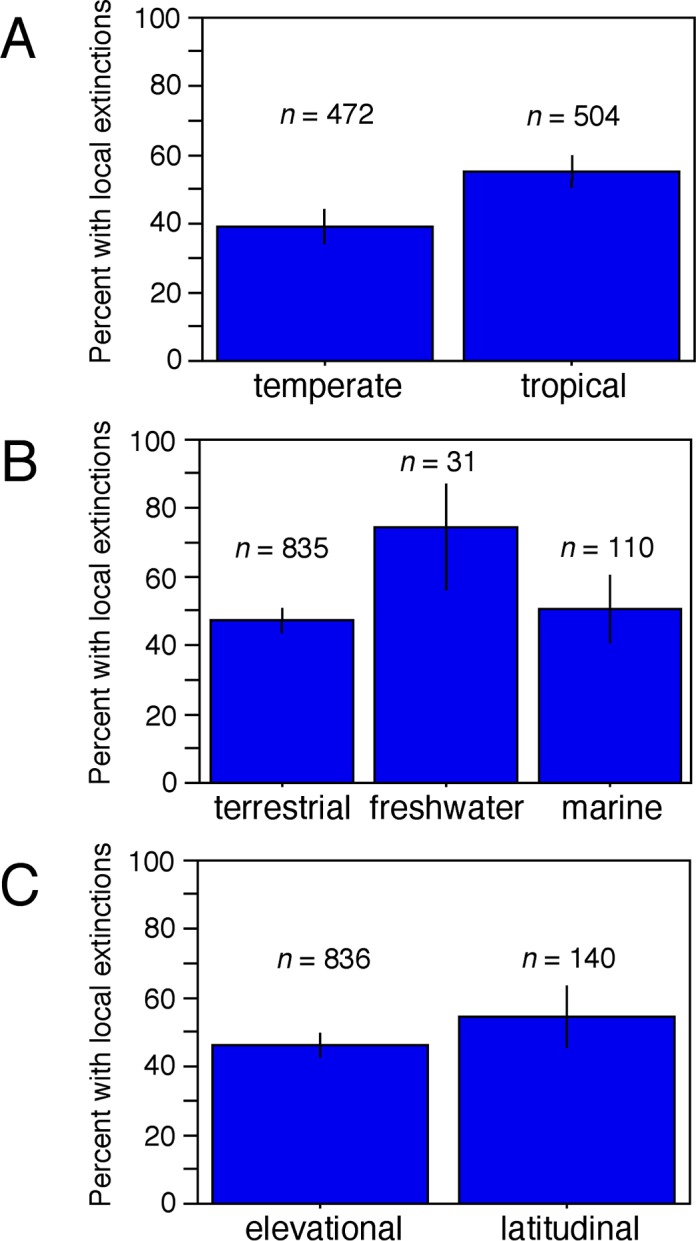
The frequency of local extinctions related to climate change across different climatic regions, habitats, and gradients. (A) Species are categorized as temperate or tropical (based on the location of the study), and the percentage of species with one or more local extinctions is shown, along with the sample sizes of species in each region. (B) Species are categorized as terrestrial, freshwater, or marine, and the frequency of species with local extinctions is shown (along with total species per habitat). (C) Species are categorized based on whether they were surveyed along elevational or latitudinal transects. Vertical lines indicate 95% confidence intervals on the estimated frequency of species with local extinctions.

Overall, the frequency of local extinctions was similar (close to 50%) across most climatic zones, habitats, gradients, and clades. Nevertheless, there were some significant differences. First, local extinctions were significantly more common in species from tropical and subtropical regions (combined and referred to as tropical hereafter for brevity) than in those from temperate regions (*p* < 0.0001; Chi-squared test, testing the assumption of equal frequencies of local extinction among species between regions; subsequent *p*-values are also from Chi-squared tests). Specifically, 54.6% of the 504 included tropical species had local extinctions, whereas only 39.2% of the 472 temperate species did (Fig 2A). The pattern was even stronger when only considering terrestrial species on elevational gradients (54.6% of 504 tropical species versus 28.2% of 301 temperate species), which applied to all plants and most animals. In part, this pattern of more frequent tropical extinction arose from a much lower frequency of extinctions for temperate plants (59.4% of 155 tropical species versus 8.6% of 105 temperate species; *p* < 0.0001). The very low frequency of temperate extinctions in plants was based on a single study from very high latitudes [19]. Nevertheless, there were also significantly more local extinctions in tropical animals (52.4% of 349 tropical species versus 38.8% of 196 temperate species; *p* = 0.0022), if one compares terrestrial species on elevational gradients. This restriction also made them more comparable to the sampled plants (all from terrestrial, elevational gradients) and still encompassed most sampled animal species (76.1%; 545 of 716 species). Across all animals, the difference was not significant (*p* = 0.2309), possibly because of the influence of temperate marine and freshwater species (see below). Among the most well-sampled groups of animals, tropical extinction was significantly more common in birds (51.4% of 109 tropical species versus 37.1% of 124 temperate species; *p* = 0.0284), but not in insects (local extinctions in 55.2% of 210 tropical species versus 59.0% of 61 temperate species; *p* = 0.6007). For other animal groups, the species sampled here were either predominantly temperate (mammals, fish, and marine invertebrates) or tropical (squamate reptiles and amphibians), and so did not allow for similar within-clade comparisons.

Overall, the frequency of climate-related local extinctions (Fig 2B) was similar in terrestrial (45.6% of 835 species) and marine environments (50.9% of 110; *p* = 0.2964). In contrast, the frequency in freshwater species was substantially higher (74.2% of 31; *p* = 0.0053 across all three habitats). However, the estimate for freshwater species was based on a single study of European fishes [17]. Comparing fish only (all temperate) also supported a significantly higher frequency of extinction in freshwater environments relative to marine environments (*p* = 0.0240; local extinctions in 47.4% of 38 marine species versus 74.2% of 31 freshwater species). All marine species included here were temperate animals, but there was no significant difference in extinction frequencies between marine and terrestrial environments when only temperate animals were compared (*p* = 0.1676; marine: 50.9% of 110 species, terrestrial: 42.9% of 226 species). Terrestrial and freshwater species remained significantly different in this more restricted comparison (*p* = 0.0011).

The frequency of local extinctions (Fig 2C) was somewhat lower for species surveyed along elevational gradients relative to those on latitudinal gradients (elevational: 45.8% of 836 species; latitudinal: 55.0% of 140 species; *p* = 0.0439). Most (78.6%) species measured along latitudinal gradients were marine (and all marine studies focused on latitudinal gradients), and all were temperate. Again, most species included here were based on studies of elevational gradients in terrestrial environments.

**Fig 3:**
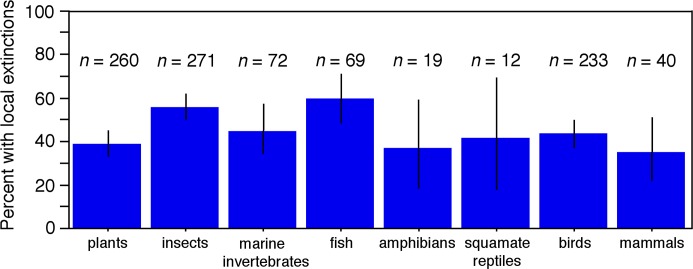
**The frequency of local extinctions related to climate change across different taxonomic groups**. The percentage of species with one or more local extinctions in each taxonomic group is shown, along with the total sample size of species surveyed in that group. For ease of presentation, four different groups of marine invertebrates (annelids, crustaceans, molluscs, and echinoderms) are shown together. Frequencies for these four groups were averaged to obtain a single value, and sample sizes of species across groups were summed. Squamate reptiles include lizards and snakes. Vertical lines indicate 95% confidence intervals on the estimated frequency of species with local extinctions.

Local extinctions were also broadly similar in frequency across taxonomic groups (Fig 3). Nevertheless, local extinctions were significantly more common (*p* = 0.0018) in animals (50.1% of 716) than plants (38.8% of 260). This difference was reduced when comparing only animals and plants on terrestrial, elevational gradients (47.3% of 556 animal species versus 38.8% of 260 plant species; *p* = 0.0236). Among these latter species, the plant–animal difference was nonsignificant for tropical species (and was actually reversed: local extinctions in 52.4% of 349 tropical animal species versus 59.4% of 155 tropical plants; *p* = 0.1500) but was strong for temperate species (38.6% of 207 temperate animal species versus 8.6% of 105 temperate plants; *p* < 0.0001).

The frequencies of local extinctions across different animal groups (Fig 3) were broadly similar to the overall value for animals (50.1%), but with higher values in insects (56.1% of 271 species; based on six studies; Table 1) and fish (59.4% of 69 species; three studies) relative to mammals (35.0% of 40 species; four studies), birds (43.8% of 233 species; five studies), amphibians (36.8% of 19 species; one study), and squamate reptiles (lizards and snakes; 41.7% of 12 species; two studies). Local extinctions were also broadly similar in frequency in various groups of marine invertebrates, including crustaceans (46.7% of 15; one study), annelids (64.5% of 31; one study), and molluscs (45.4% of 22; two studies). The frequency in echinoderms was lower (25.0%; one study) but was based on a very small sample size (4 species).

Results were generally similar using both general linear models (GLMs; see below) and general linear mixed models (GLMMs; see next paragraph). GLM results are given in full in S2 Appendix and are summarized here. Simultaneously including all 976 species and most variables (habitat [terrestrial versus freshwater versus marine], climatic regions [tropical versus temperate], taxonomic group [plants versus animals], survey type [latitudinal versus elevational], and study dates [start date, end date, and duration in between]) showed that most variables had significant effects on the frequency of extinction, except for the study dates. There were strong effects of habitat and climate (*p* < 0.00001) but weaker effects of taxonomic group (*p* = 0.0246). Results were similar when excluding study dates and taxonomic group. Including geographic regions showed that most regions had no significant effect (except for Madagascar and South America). Given that Madagascar and South America were represented by one study each, these region effects were not considered further. Furthermore, the effects of climatic region, habitat, taxonomic group, and survey type remained significant when geographic regions were included. Comparing species only on terrestrial elevational gradients (805 species in total) further confirmed the significant effects of climate and taxonomic group. Similarly, considering plants only (260 species) also confirmed the significant effects of climatic region. Considering only terrestrial animals on elevational gradients (545 species) showed a significant effect of climate (*p* = 0.0023) after removing study dates, which had no significant effect. Considering birds alone (233 species) and including climatic region, survey type, and study dates showed that climatic region, survey type, start date, and end date had significant effects. For insects (271 species), when including climatic region, study dates, and survey type, no variables were significant. For fish (69 species), a model including habitat (freshwater versus marine), study dates, and survey type showed that no variables were significant. However, habitat was significant if other variables were removed. Similarly, for temperate animals (367 species), a model including habitat, survey type, and study dates showed that only habitat and survey type were significant. Comparison of plants and animals on terrestrial elevational gradients (including study dates) showed that extinction is significantly different between temperate plants and animals (more common in animals), but not between tropical ones. Across animals, the effects of taxonomic group were limited and depended on the other variables included. If only taxonomic groups and study dates were included, then annelids, fish, and insects showed significantly more extinction (*p* = 0.03–0.05). Including habitat and survey type (and removing study dates) showed stronger effects in fish and annelids (as well as in crustaceans and molluscs), but not in insects.

Results were also broadly similar using GLMMs, with study identity included as a random effect. Results are summarized below and given in full in S3 Appendix. The impacts of study dates were somewhat counterintuitive (and rarely significant), and analyses including them sometimes failed. When most variables were included (habitat, climatic region, taxonomic group [plant versus animal], survey type, and study dates), all variables were significant except for study dates and taxonomic group, with strong effects of habitat, climatic region, and survey type. When study dates were removed, only habitat and survey type were significant. When geographic regions were included (and study dates excluded), only South America had a significant effect, and habitat, taxonomic group, and climatic region were significant or marginally significant. Comparing tropical and temperate species on terrestrial, elevational gradients showed significant effects of climatic region (*p* = 0.0017) and taxonomic group (*p* = 0.0119), but not of study dates. When study dates were removed, no variables were significant. Plants alone showed a significant effect of climatic region (*p* < 0.0001), but analyses failed if study dates were included. Animals on terrestrial, elevational gradients showed no significant effect of climatic region (again, study dates had to be excluded). Considering birds alone showed no significant effect of climate but a significant effect of survey type (excluding study dates). Insects showed no significant effects of climate or survey type, regardless of whether study dates were included. Analyses of fish failed unless study dates and survey type were excluded, but habitat alone (marine versus freshwater) had a significant effect (*p* = 0.0265). Analyses of temperate animals (367 species) including habitat, survey type, and study dates showed only habitat type as significant (*p* = 0.0307), but excluding study dates showed significant effects of habitat and survey type. Comparing only temperate plants and animals showed a significant effect of taxonomic group, when study dates were included (*p* = 0.0116) or excluded (*p* = 0.0005; study dates had no significant effect). In contrast, there was no significant effect of taxonomic group when comparing tropical plants and animals (504 species total; excluding study dates). Analyses of animals alone showed no significant effect of taxonomic group.

In summary, several patterns emerged as significant across all (or most) analyses. First, there were significant effects of climatic region overall, with extinction more common in tropical regions. This was present in plants across all analyses and generally present in animals. Animals showed significantly more extinction than plants overall and when comparing temperate, but not tropical, species. There were significant effects of habitat on animals overall (higher extinction in freshwater), even when considering fish alone. Finally, GLM analyses showed some effects of taxonomic groups across animals (with higher extinction in fish and annelids) and possibly in insects, molluscs, and crustaceans. The GLMM analyses did not show these group effects, possibly because many animal groups are included based on a single study.

## Discussion

The results of this study show that local extinctions (inferred to be related to climate change) are already widespread and have occurred in hundreds of species. Roughly half of the 976 species that were surveyed for range shifts showed evidence of local extinctions (47%). This proportion was surprisingly similar across diverse climatic regions, habitats, and taxonomic groups. The results here suggest that even the modest changes in climate that have occurred so far are enough to drive local populations in many species to extinction. The results here also suggest that local populations in many species cannot shift their climatic niches rapidly enough to prevent extinction. This pattern of widespread local extinction seems likely to become even more prevalent as the global climate warms further (by roughly 2 to 5-fold [4]) in the next several decades.

The results here showed generally similar patterns of local extinction across climatic zones, habitats, and clades. Nevertheless, most analyses showed that local extinctions were significantly more common in tropical species (Fig 2A), in freshwater species (Fig 2B), and in animals. A greater impact of climate change on tropical species has been predicted by several authors (e.g., [40–42]). This prediction is related to the narrower climatic niche widths for temperature-related variables in tropical species that are associated with reduced temperature seasonality in the tropics (e.g., [43,44]) and lower rates of temperature-related climatic niche change in tropical species (e.g., [42]). The results here provide support for this prediction based on documented local extinctions that have already occurred: species in tropical regions had local extinctions more frequently than those in temperate regions (54.6% versus 39.2%), especially when species were compared on terrestrial, elevational gradients (54.6% versus 28.2%). This pattern was strongest in plants and when animals were compared on terrestrial elevational gradients. Overall, these results further support the idea that the negative impacts of climate change on biodiversity are more frequent (per species) in tropical regions [40–42], where biodiversity is highest.

Climate-related local extinctions were also similar in frequency in marine and terrestrial species (Fig 2B) but were more common in freshwater species (although freshwater habitats were represented by a single study). Freshwater species may be especially susceptible to changes in precipitation patterns (e.g., drought), which can substantially alter or eliminate their habitats (e.g., [45]), quickly resulting in local extinction. In contrast, marine species may experience less impact from changes in precipitation. Furthermore, they may be buffered from temperature changes because they can potentially adjust the temperatures that they experience by movement within the water column (more so than is possible for most freshwater species; [46,47]).

The frequency of local extinctions was also broadly similar across diverse taxonomic groups (~35%–60%; Fig 3), including plants, insects, fish, amphibians, squamate reptiles, endothermic vertebrates (birds and mammals), and many marine invertebrates (annelids, crustaceans, and molluscs). However, local extinctions were significantly more common in animals than plants (and animals are far more species-rich than plants). They were also relatively common in insects (the most species-rich group of animals) and fish (the most species-rich group of vertebrates). Local extinctions were not particularly common in amphibians (36.7%) or squamate reptiles (41.7%), although both groups were included here based primarily on one study [32]. Nevertheless, both groups appear to have been strongly impacted by climate change overall. For example, many amphibian species have undergone sharp declines and global extinctions, many of which are thought to be caused by an interaction between climate change and an infectious disease (chytrid fungus; [48]). However, these chytrid studies were not included here because they were not focused on surveying warm-edge populations over time. Similarly, local extinctions related to climate change have been documented in many lizard species [49]. Again, these were not included here because they were not based on a systematic survey of warm-edge populations. Nevertheless, if the species studied by Sinervo et al. [49] were included here, the frequency of local extinctions in squamates would go from 41.7% (of 12 species) to 77.4% (of 124 species), but with the caveat that their study focused on documenting local extinctions and so might overestimate this frequency. It should also be noted that the well-publicized declines in amphibian populations globally are not necessarily inconsistent with the frequency of local extinction observed here. For example, a global assessment of amphibian populations [50] noted declines in 43% of amphibian species (compare to the 47% of all species here with local extinctions and the 37% for amphibians), but these declines also included those unrelated to climate change (e.g., habitat destruction and overexploitation). Thus, the frequency of climate-related declines here is not necessarily an underestimation relative to the declines documented by the global amphibian assessment [50].

A major conclusion of this study is that populations of many species are already unable to undergo niche shifts that are fast enough to prevent local extinction from climate change. The rate is emphasized here because even if the absolute amount of niche change needed to avoid extinction might be attainable, it might require more time to achieve than is allowed by the rapid pace of anthropogenic climate change. Given this result, and that climate is predicted to change even further in the near future, the persistence of many species might depend largely on their ability to successfully shift their geographic ranges to higher latitudes or elevations and remain within their original climatic niche. Indeed, the summary here shows numerous instances of cool-edge expansions (in 367 of 904 species, with cool edges that were stable in 371 others and contracted in 166 others).

Unfortunately, these movements may be impeded for many species by one or more factors. First, human impacts may prevent species from successfully dispersing (including agriculture, roads, and urbanization), or these human impacts may simply leave them no habitat to disperse to (e.g., [51,52]). Second, many species are already confined to islands, peninsulas, and mountaintops, where dispersal to higher latitudes or elevations may not be possible (e.g., [53]). Third, even if dispersal is unimpeded by human or natural barriers, it may simply occur too slowly to allow species to remain within their climatic niche (e.g., [54,55]).

The combination of these potential limits to dispersal and the widespread local extinctions documented here is troubling. However, the results here do not rule out the possibility that rapid niche shifts will occur in some populations of many species in the future, preventing global extinctions. Indeed, roughly half of the species surveyed showed no local extinctions, and most species had some populations that persisted locally (but again, this is under the limited climate change that has already occurred). The future persistence of species will depend on many factors [6,8], including rates and patterns of climate change at each location, dispersal, niche shifts, local climatic microrefugia [56], and the contribution of population-level niche width to species-level niche width (e.g., whether species are broadly tolerant or locally specialized to different climatic conditions across their ranges [44]). Most importantly, I suggest that the patterns of present-day local extinctions obtained from range-shift studies should be part of the evidence used to predict species persistence in the future.

There are several potential sources of bias that may have influenced some aspects of these results but should not overturn the major conclusions. First, “local extinction” means that individuals of a given species are entirely absent from a location that they previously occupied. However, it can be difficult to distinguish between extinction and a substantial decline in abundance that causes the species to go undetected at a given location (e.g., [57]), and studies did not necessarily provide statistical evidence for the absence of a species at a site. Here, the estimates of previous researchers were used, and it was assumed that they adequately documented local absences (otherwise, their estimates of range shifts would also be erroneous). Furthermore, strong declines that make a species undetectable at a given site might soon lead to local extinction. Second, there may be a bias in terms of unpublished results. Specifically, some researchers who monitored the warm edge of a population but failed to find any changes associated with climate change may not have published their negative results. Such a reporting bias would lead to overestimating the proportion of species experiencing local extinction in this study. Nevertheless, local extinctions were still documented in hundreds of species across regions and clades, even if there are hundreds of additional species in which these local extinctions did not occur. Additionally, numerous species (*n* = 171) showed evidence of a cool-edge expansion without a corresponding contraction in the warm edge. Thus, a species can undergo a range shift but without local extinction, which should limit this source of publication bias. Third, it was assumed that previous researchers correctly associated the patterns that they observed with climate change. In theory, other factors such as overharvesting or habitat destruction may have contributed to the observed local extinctions in some cases (e.g., [21]). Again, the analyses here primarily assume that the main conclusions of these previous studies were not erroneous.

Finally, despite the widespread pattern of warm-edge contractions and local extinctions, 521 species showed no local extinctions at the warm edge, indicating that they have successfully persisted in the face of the climate change that has occurred so far. However, even these species might still go globally extinct when global climate changes further. Additionally, contrary to the overall trend, 54 species were documented here as having expansions at both their warm edge and their cool edge (6.0% of 904 species with data on both cool and warm edges). One scenario by which this may occur is if cool-edge limits are set by colder temperatures (allowing expansion as global climate warms) and warm-edge limits are set by low precipitation (allowing warm-edge expansion), given that precipitation may increase in some areas because of climate change [4]. Indeed, some studies have found evidence for warm-edge expansions through this mechanism [58]. It is also important to note that local extinctions related to climate change need not be confined to the warm edge of the species range and so might actually be underestimated here. For example, there could be climate-related local extinctions far from the warm edge that are associated with certain microclimates (e.g., equatorially facing slopes at the cool edge of a species range; [59]).

In summary, the results here show that widespread local extinctions (seemingly related to climate change) have already occurred in hundreds of species, with broadly similar patterns of extinction across diverse clades, habitats, and climatic regions. Importantly, levels of climate change so far are limited relative to those generally predicted for the next 100 years [4]. The results here suggest that many species are unable to shift their niches rapidly enough to prevent local extinction. This inference of climate change outpacing niche change supports predictions from other sources, including transplant experiments in plants [60], phylogenetic analyses of rates of niche change in plants and animals [42,61,62], and projections based on selection, heritability, and temperature tolerances in lizards [49]. Local extinctions from climate change might also impact species that many human populations depend on for food, such as grasses (e.g., wheat, rice, and corn [62]). More generally, this study demonstrates that analyses of range shifts can provide extensive data on local extinctions related to climate change that have already occurred. These local extinctions offer a potentially important but underutilized source of information for the challenging task of predicting patterns of species survival and extinction in the future.

## Materials and Methods

### Selection of Studies

Web of Science searches were initially conducted from December 2014 to April 2015 using the Boolean search terms Topic = (global warming OR climate change) AND Topic = (local extinction OR range contraction OR range shift). A second Web of Science search was conducted between April 2015 and May 2015 to identify additional studies potentially missed by the first set of keywords, using the search terms TS = (global warm* OR climate change) AND TS = (extinction* OR contraction* OR range shift*), excluding results from TS = (global warming OR climate change) AND TS = (local extinction OR range contraction OR range shift). Each set of Web of Science results was sorted by relevance and then binned into subsets of 50. Searching was ceased when less than 1 in 50 studies per subset was relevant (see below for criteria). Finally, a third Web of Science search was performed on 1 March 2016 to find more recently published studies. This third search used the keywords TS = (global warm* OR climate change) AND TS = (extinction* OR contraction* OR range shift*). A total of 1,530 results were found in this third search. Results were sorted by relevance, and the first 300 (~20%) were examined. The last 40 of these 300 included no relevant studies.

Some additional studies were also found that were listed as references in the papers identified by these initial Web of Science searches. The reference list was also checked against a recent review study [11], which also conducted thorough searches of the literature on climate-related range shifts. Three studies were added from that survey which were not initially included here. Finally, several relevant studies were also found in the survey of Gibson-Renemer et al. [63], which had similar rules for inclusion of studies. Although those authors did not conduct a systematic search of the literature (as done here), they nevertheless included five studies not found in the searches described above. These were also added here.

In theory, the fact that “extinction” and “contraction” were included as keywords might have biased the results to include more papers documenting local extinctions and range contractions than would be obtained from a search of range-shift studies that excluded these as keywords (possibly leading to overestimation of the frequency of local extinctions). However, this seems unlikely in practice. First, these were included as “or” keywords, along with “range shifts.” Examining the keywords and titles of the 27 selected papers showed that most were focused on overall range shifts, with no mention of local extinction (extinction or extirpation are mentioned in the titles of only 4 of 27 studies and as keywords in only 4 of the 21 studies with keywords; “contraction” is mentioned in only 1). Furthermore, the fact that the survey results here were checked against another recent review on range shifts [11], and that three missing studies were added, also makes this potential bias seem unlikely. In other words, if many range-shift studies were missed because of this bias, they should have been added at that point.

Overall, these searches were extensive but may not be truly exhaustive. Regardless, many studies were found that documented local extinctions, and finding more studies that did so would not overturn this main conclusion.

Studies were included that monitored one or more populations at the warm edge of a species’ range (the edge that is lower in elevation or closer to the equator) over a relatively long time span. Studies were only included that spanned an interval of at least 10 years. The mean study duration was ~50 years (range = 14 to 159; Table 1). Studies were included that related their findings on range shifts to climate change through an explicit statistical analysis (but noting that these inferences could still be incorrect, for example, if other factors instead of climate change caused local extinctions of a particular species). The included studies all documented populations along elevational or latitudinal transects at two or more discrete time points.

Some recent studies have inferred climate-related range shifts based on overall trends in latitudinal and elevational distributions across a large number of localities over time, rather than systematically resurveying specific localities at different time points (e.g., [64]). These studies are valuable for documenting range shifts in general but were excluded here, since they do not unambiguously represent local extinctions (because the overall patterns described might be driven solely by range expansions instead).

### Categorizing Species

Studies that documented warm-edge range contractions (and that were linked to climate change by the authors of the original studies) were considered evidence of climate-associated local extinction, regardless of changes at the cool edge. Studies differed in whether they reported changes at the population level (e.g., [28,37]) or species level (e.g., [33]). The analysis here was conducted at the species level. Therefore, if populations of the same species differed in the pattern of their range shifts, the species was categorized as showing evidence of local extinction if at least one population did so.

Most species were included in only one study. However, the plant species *Anthoxanthum odoratum* was included by both Angelo and Daehler [13] (in Hawaii) and Felde et al. [19] (in Europe). However, since this species is not native to Hawaii, it was excluded from the dataset of Angelo and Daehler [13], along with all other nonnative species in that study.

For each study, it was noted whether the range shifts were elevational or latitudinal, as well as the general habitat of the organisms (i.e., terrestrial, freshwater, or marine), the higher taxa to which they belonged, the specific geographic location of the study, and whether the species occurred in a tropical or subtropical region (arbitrarily defined as within 35° of the equator) or in a temperate region (>35°). Species were assigned to these climatic regions based solely on the location where they were surveyed, rather than on their overall geographic range. Species were also assigned to taxonomic categories, including plants, insects, fish, amphibians, birds, mammals, and squamate reptiles (i.e., lizards and snakes), as well as marine annelids, crustaceans, echinoderms, and molluscs. The beginning and end dates of the study were also noted (e.g., the date of the initial survey and the subsequent resurvey) and were used to estimate the duration of the study. Some studies provided a range of dates for the start and/or end date. In these cases, the midpoint of each range of dates was used to estimate the start, end, and duration (Table 1). Data for all species are provided in S1 Appendix.

The studies included (Table 1) spanned many geographic regions (e.g., North America, South America, Europe, Asia, and Oceania). Many studies were conducted in North America (*n* = 13; here extending to Central America) and Europe (*n* = 8), but the actual number of species sampled was more broadly distributed among regions (e.g., Asia = 332; Europe = 268; Madagascar = 30; Oceania = 58; North America = 233; and South America = 55). Africa and Australia were not represented, although nearby Madagascar and New Guinea were. The numbers of temperate and tropical species included were nearly equal. Further, there was no clear hypothesis for why particular continents alone should be an important factor influencing the frequency of local extinctions (e.g., separate from temperate versus tropical effects).

### Statistical Analyses

Chi-squared analyses were initially used to compare the proportion of climate-associated local extinctions across some categories (i.e., tropical versus temperate; freshwater versus marine versus terrestrial; and latitudinal versus elevational gradients), testing the null hypothesis that frequencies of local extinction were equal between these categories. A series of analyses were conducted to assess whether frequencies of local extinction were higher in tropical regions relative to temperate regions, after accounting for the potential influence of different habitats, gradients, and clades (see Results). Similar analyses were conducted to assess the impacts of different habitats and clades (i.e., plants versus animals). However, potential analyses were restricted by the available data. For example, it was not possible to compare the effect of tropical versus temperate climates on marine or freshwater organisms, since only temperate marine and freshwater species were included here. For this reason, different sets of analyses were conducted for each question.

These analyses were then repeated using GLMs and GLMMs, both in R. These analyses were implemented treating the presence of warm-edge local extinction in a species as the binomial, dependent variable. GLMM analyses were conducted using the R package *lme4* [65]. GLMM analyses treated the study (from which the species data were obtained) as the random variable and the other variables as the fixed variables. GLM and GLMM analyses initially included all species and all or most variables and were then restricted to smaller sets of species (and variables) to test additional hypotheses and reduce potentially confounding effects (as in the Chi-squared analyses).

Phylogenetic information was not incorporated here, since phylogenies and comparable branch lengths spanning all the included species were not available (especially species-level phylogenies for fish, insects, plants, and marine invertebrates). Nevertheless, some analyses were conducted to assess patterns within and between clades (see Results).

## Supporting Information

S1 Appendix Data for the 976 species used in this study. (XLSX)

S2 Appendix Results of GLM analyses, showing variable coefficients. (DOC)

S3 Appendix Results of GLMM analyses, showing variable coefficients. (DOC)

## Abbreviations

GLM: general linear model
GLMM: general linear mixed model
