# Supplementary material for: Climate-Related Local Extinctions Are Already Widespread among Plant and Animal Species
Source: PLoS Biol. 2016 Dec 8;14(12):e2001104. doi: 10.1371/journal.pbio.2001104 (PMC5147797; doi:10.1371/journal.pbio.2001104)
Supplement: S2 Appendix — (DOC) [file pbio.2001104.s002.doc]

Appendix S2. Results of GLM analyses, showing variable coefficients.

Appendix S2, Table 1. Results of GLM analyses incuding all 976 species, testing effects of habitat, climatic region, the start date, end date, and duration of the study, taxonomic group (plants vs. animals), and whether the species were surveyed on a latitudinal or elevational gradient, on the frequency of local extinction.

|  | Estimate | Standard error | z-value | p-value |
| --- | --- | --- | --- | --- |
| (Intercept) | -76.78159 | 37.37440 | -2.054 | 0.0399 |
| **HabitatMarine** | **-2.67899** | **0.62173** | **-4.309** | **1.64e-05** |
| **HabitatTerrestrial** | **-1.77474** | **0.44242** | **-4.011** | **6.04e-05** |
| **Climatic.regionTropical** | **0.89783** | **0.18611** | **4.824** | **1.40e-06** |
| Start | -0.02631 | 0.05208 | -0.505 | 0.6134 |
| End | 0.06509 | 0.05493 | 1.185 | 0.2360 |
| Duration | -0.02390 | 0.05205 | -0.459 | 0.6461 |
| **GroupPlant** | **-0.65369** | **0.29079** | **-2.248** | **0.0246** |
| **Latitude.or.elevationLatitude** | **1.76742** | **0.42634** | **4.146** | **3.39e-05** |

Appendix S2, Table 2. Results of GLM analyses including 976 species, testing effects of habitat, climatic region, taxonomic group (plants vs. animals), and whether the species were surveyed on a latitudinal or elevational gradient, on the frequency of local extinction.

|  | Estimate | Standard error | z-value | p-value |
| --- | --- | --- | --- | --- |
| (Intercept) | 1.0561 | 0.4105 | 2.573 | 0.0101 |
| **HabitatMarine** | **-2.6902** | **0.6185** | **-4.350** | **1.36e-05** |
| **HabitatTerrestrial** | **-1.8793** | **0.4330** | **-4.341** | **1.42e-05** |
| **Climatic.regionTropical** | **1.1083** | **0.1566** | **7.078** | **1.47e-12** |
| **GroupPlant** | **-0.3291** | **0.1586** | **-2.075** | **0.0379** |
| **Latitude.or.elevationLatitude** | **1.6706** | **0.4216** | **3.963** | **7.41e-05** |

Appendix S2, Table 3. Results of GLM analyses including all 976 species, testing effects of habitat, climatic region, and whether the species were surveyed on a latitudinal or elevational gradient on the frequency of local extinction.

|  | Estimate | Standard error | z-value | p-value |
| --- | --- | --- | --- | --- |
| (Intercept) | 1.0561 | 0.4105 | 2.573 | 0.0101 |
| **HabitatMarine** | **-2.7996** | **0.6164** | **-4.542** | **5.58e-06** |
| **HabitatTerrestrial** | **-1.9887** | **0.4300** | **-4.625** | **3.74e-06** |
| **Climatic.regionTropical** | **1.1157** | **0.1562** | **7.143** | **9.15e-13** |
| **Latitude.or.elevationLatitude** | **1.7799** | **0.4185** | **4.253** | **2.11e-05** |

Appendix S2, Table 4. Results of GLM analyses including all 976 species, testing effects of habitat, climatic region, taxonomic group (plants vs. animals), whether the species were surveyed on a latitudinal or elevational gradient, and different geographic regions, on the frequency of local extinction.

|  | Estimate | Standard error | z-value | p-value |
| --- | --- | --- | --- | --- |
| (Intercept) | 0.81434 | 0.63081 | 1.291 | 0.196724 |
| **HabitatMarine** | **-2.64559** | **0.61905** | **-4.274** | **1.92e-05** |
| **HabitatTerrestrial** | **-1.72812** | **0.45213** | **-3.822** | **0.000132** |
| **Climatic.regionTropical** | **1.42197** | **0.43889** | **3.240** | **0.001196** |
| **GroupPlant** | **-0.53631** | **0.18694** | **-2.869** | **0.004118** |
| **Latitude.or.elevationLatitude** | **1.68832** | **0.43079** | **3.919** | **8.89e-05** |
| Geographic.regionEurope | 0.24171 | 0.47901 | 0.505 | 0.613834 |
| **Geographic.regionMadagascar** | **-1.05474** | **0.40168** | **-2.626** | **0.008645** |
| Geographic.regionNorth America | 0.06113 | 0.40213 | 0.152 | 0.879181 |
| Geographic.regionOceania (Hawaii) | 0.02812 | 1.01272 | 0.028 | 0.977849 |
| Geographic.regionOceania (New Guinea) | 0.54163 | 0.33799 | 1.602 | 0.109046 |
| **Geographic.regionSouth America** | **-1.39917** | **0.32550** | **-4.299** | **1.72e-05** |

Appendix S2, Table 5. Results of GLM analyses including only terrestrial species surveyed on elevational gradients (805 species), testing effects of climatic region, the start date, end date, and duration of the study, and taxonomic group (plants vs. animals) on the frequency of local extinction.

|  | Estimate | Standard error | z-value | p-value |
| --- | --- | --- | --- | --- |
| (Intercept) | -104.38750 | 43.16155 | -2.419 | 0.01558 |
| **Climatic.regionTropical** | **0.83631** | **0.19485** | **4.292** | **1.77e-05** |
| Start | -0.02855 | 0.05206 | -0.548 | 0.58346 |
| End | 0.08014 | 0.05597 | 1.432 | 0.15215 |
| Duration | -0.02233 | 0.05204 | -0.429 | 0.66788 |
| GroupPlant | -1.00764 | 0.32333 | -3.116 | 0.00183 |

Appendix S2, Table 6. Results of GLM analyses including only terrestrial species surveyed on elevational gradients (805 species), testing effects of climatic region and taxonomic group (plants vs. animals) on the frequency of local extinction.

|  | Estimate | Standard error | z-value | p-value |
| --- | --- | --- | --- | --- |
| (Intercept) | -0.8233 | 0.1378 | -5.976 | 2.28e-09 |
| **Climatic.regionTropical** | **1.1083** | **0.1566** | **7.078** | **1.47e-12** |
| **GroupPlant** | **-0.3291** | **0.1586** | **-2.075** | **0.0379** |

Appendix S2, Table 7. Results of GLM analyses including only plant species (260 species), testing effects of climatic region, and the start date, end date, and duration of the study on the frequency of local extinction. The results for duration are unavailable because of a singularity in the data (NA).

|  | Estimate | Standard error | z-value | p-value |
| --- | --- | --- | --- | --- |
| (Intercept) | -1.376e+02 | 6.905e+02 | -0.199 | 0.842 |
| **Climatic.regionTropical** | **2.589e+00** | **6.391e-01** | **4.051** | **5.1e-05** |
| Start | -3.337e-03 | 7.351e-03 | -0.454 | 0.650 |
| End | 7.049e-02 | 3.499e-01 | 0.201 | 0.840 |
| Duration | NA | NA | NA | NA |

Appendix S2, Table 8. Results of GLM analyses including only plant species (260 species), testing effects of climatic region on the frequency of local extinction.

|  | Estimate | Standard error | z-value | p-value |
| --- | --- | --- | --- | --- |
| (Intercept) | -2.3671 | 0.3486 | -6.790 | 1.12e-11 |
| **Climatic.regionTropical** | **2.7458** | **0.3851** | **7.131** | **9.98e-13** |

Appendix S2, Table 9. Results of GLM analyses including only terrestrial animal species surveyed on elevational gradients (545 species), testing effects of climatic region, the start date, end date, and duration of the study on the frequency of local extinction.

|  | Estimate | Standard error | z-value | p-value |
| --- | --- | --- | --- | --- |
| (Intercept) | -165.86837 | 45.98262 | -3.607 | 0.00031 |
| Climatic.regionTropical | -0.11358 | 0.24131 | -0.471 | 0.63788 |
| Start | -0.01230 | 0.05122 | -0.240 | 0.81014 |
| End | 0.09505 | 0.05619 | 1.692 | 0.09073 |
| Duration | -0.01419 | 0.05108 | -0.278 | 0.78118 |

Appendix S2, Table 10. Results of GLM analyses including only terrestrial animal species surveyed on elevational gradients (545 species), testing effects of climatic region on the frequency of local extinction.

|  | Estimate | Standard error | z-value | p-value |
| --- | --- | --- | --- | --- |
| (Intercept) | -0.4568 | 0.1466 | -3.116 | 0.00184 |
| **Climatic.regionTropical** | **0.5543** | **0.1816** | **3.052** | **0.00227** |

Appendix S2, Table 11. Results of GLM analyses including only bird species (233 species), testing effects of climatic region, the start date, end date, and duration of the study on the frequency of local extinction.

|  | Estimate | Standard error | z-value | p-value |
| --- | --- | --- | --- | --- |
| (Intercept) | -1.432e+03 | 2.898e+02 | -4.941 | 7.79e-07 |
| **Climatic.regionTropical** | **-8.912e+00** | **1.835e+00** | **-4.856** | **1.20e-06** |
| **Start** | **-8.177e-02** | **2.408e-02** | **-3.396** | **0.000684** |
| **End** | **7.964e-01** | **1.674e-01** | **4.757** | **1.97e-06** |
| Duration | NA | NA | NA | NA |

Appendix S2, Table 12. Results of GLM analyses including only bird species (233 species), testing effects of climatic region, the start date, end date, and duration of the study, and the type of survey (elevational vs. latitudinal) on the frequency of local extinction.

|  | Estimate | Standard error | z-value | p-value |
| --- | --- | --- | --- | --- |
| (Intercept) | -1.352e+03 | 2.916e+02 | -4.637 | 3.53e-06 |
| **Climatic.regionTropical** | **-7.644e+00** | **1.887e+00** | **-4.051** | **5.09e-05** |
| **Start** | **-9.754e-02** | **2.468e-02** | **-3.952** | **7.75e-05** |
| **End** | **7.718e-01** | **1.680e-01** | **4.594** | **4.35e-06** |
| Duration | NA | NA | NA | NA |
| **Latitude.or.elevationLatitude** | **2.584e+00** | **9.427e-01** | **2.741** | **0.00612** |

Appendix S2, Table 13. Results of GLM analyses including only insect species (271 species), testing effects of climatic region, the start date, end date, and duration of the study, and the type of survey (elevational vs. latitudinal) on the frequency of local extinction.

|  | Estimate | Standard error | z-value | p-value |
| --- | --- | --- | --- | --- |
| (Intercept) | 452.3807 | 476.0796 | 0.950 | 0.342 |
| Climatic.regionTropical | -0.6357 | 1.1930 | -0.533 | 0.594 |
| Start | 0.1326 | 0.2604 | 0.509 | 0.611 |
| End | -0.3581 | 0.3694 | -0.969 | 0.332 |
| Duration | 0.1570 | 0.2664 | 0.589 | 0.556 |
| Latitude.or.elevationLatitude | 13.6816 | 624.1942 | 0.022 | 0.983 |

Appendix S2, Table 14. Results of GLM analyses including only fish species (69 species), testing effects of habitat (freshwater vs. marine), the start date, end date, and duration of the study, and the type of survey (elevational vs. latitudinal) on the frequency of local extinction.

|  | Estimate | Standard error | z-value | p-value |
| --- | --- | --- | --- | --- |
| (Intercept) | 90.52869 | 165.06210 | 0.548 | 0.583 |
| HabitatMarine | -1.86698 | 1.40641 | -1.327 | 0.184 |
| Start | -0.04505 | 0.08311 | -0.542 | 0.588 |
| End | NA | NA | NA | NA |
| Duration | NA | NA | NA | NA |
| Latitude.or.elevationLatitude | NA | NA | NA | NA |

Appendix S2, Table 15. Results of GLM analyses including only fish species (69 species), testing effects of habitat (freshwater vs. marine only) on the frequency of local extinction.

|  | Estimate | Standard error | z-value | p-value |
| --- | --- | --- | --- | --- |
| (Intercept) | 1.0561 | 0.4105 | 2.573 | 0.0101 |
| **HabitatMarine** | **-1.1614** | **0.5235** | **-2.219** | **0.0265** |

Appendix S2, Table 16. Results of GLM analyses including only fish species (69 species), testing the effects of habitat (freshwater vs. marine), the start date, end date, and duration of the study on the frequency of local extinction.

|  | Estimate | Standard error | z-value | p-value |
| --- | --- | --- | --- | --- |
| (Intercept) | 90.52869 | 165.06210 | 0.548 | 0.583 |
| HabitatMarine | -1.86698 | 1.40641 | -1.327 | 0.184 |
| Start | -0.04505 | 0.08311 | -0.542 | 0.588 |
| End | NA | NA | NA | NA |
| Duration | NA | NA | NA | NA |

Appendix S2, Table 17. Results of GLM analyses including only temperate animal species (367 species), testing effects of habitat (terrestrial, freshwater vs. marine), the start date, end date, and duration of the study on the frequency of local extinction.

|  | Estimate | Standard error | z-value | p-value |
| --- | --- | --- | --- | --- |
| (Intercept) | -9.541e+01 | 4.529e+01 | -2.107 | 0.0351 |
| HabitatMarine | -7.543e-01 | 4.581e-01 | -1.647 | 0.0996 |
| HabitatTerrestrial | -4.849e-01 | 4.627e-01 | -1.048 | 0.2947 |
| Start | 4.239e-04 | 5.221e-02 | 0.008 | 0.9935 |
| End | 4.784e-02 | 5.681e-02 | 0.842 | 0.3997 |
| Duration | -1.682e-02 | 5.211e-02 | -0.323 | 0.7469 |

Appendix S2, Table 18. Results of GLM analyses including only temperate animal species (367 species), testing effects of habitat (terrestrial, freshwater vs. marine) and type of survey (elevational vs. latitudinal) on the frequency of local extinction.

|  | Estimate | Standard error | z-value | p-value |
| --- | --- | --- | --- | --- |
| (Intercept) | 1.0561 | 0.4105 | 2.573 | 0.010087 |
| **HabitatMarine** | **-2.3237** | **0.6205** | **-3.745** | **0.000181** |
| **HabitatTerrestrial** | **-1.5128** | **0.4359** | **-3.471** | **0.000519** |
| **Latitude.or.elevationLatitude** | **1.3041** | **0.4245** | **3.072** | **0.002128** |

Appendix S2, Table 19. Results of GLM analyses including only temperate animal species (367 species), testing effects of habitat (terrestrial, freshwater vs. marine), survey type, and the start date, end date, and duration of the study on the frequency of local extinction.

|  | Estimate | Standard error | z-value | p-value |
| --- | --- | --- | --- | --- |
| (Intercept) | -1.080e+02 | 4.558e+01 | -2.369 | 0.01784 |
| **HabitatMarine** | **-1.750e+00** | **6.413e-01** | **-2.728** | **0.00637** |
| HabitatTerrestrial | -6.792e-01 | 4.693e-01 | -1.447 | 0.14778 |
| **Latitude.or.elevationLatitude** | **9.976e-01** | **4.516e-01** | **2.209** | **0.02718** |
| Start | -3.016e-03 | 5.147e-02 | -0.059 | 0.95328 |
| End | 5.751e-02 | 5.625e-02 | 1.023 | 0.30652 |
| Duration | -1.743e-02 | 5.136e-02 | -0.339 | 0.73434 |

Appendix S2, Table 20. Results of GLM analyses comparing temperate animal and plant species on terrestrial, elevational gradients (301 species total), including study start date, end date, and duration on the frequency of local extinction.

|  | Estimate | Standard error | z-value | p-value |
| --- | --- | --- | --- | --- |
| (Intercept) | -1.434e+02 | 6.415e+01 | -2.236 | 0.02536 |
| **GroupPlant** | **-2.130e+00** | **6.600e-01** | **-3.227** | **0.00125** |
| Start | -8.903e-03 | 5.135e-02 | -0.173 | 0.86235 |
| End | 8.057e-02 | 6.139e-02 | 1.312 | 0.18939 |
| Duration | -1.542e-02 | 5.119e-02 | -0.301 | 0.76327 |

Appendix S2, Table 21. Results of GLM analyses comparing the frequency of local extinction in tropical animal and plant species on terrestrial, elevational gradients (504 species total), including study start date, end date, and duration.

|  | Estimate | Standard error | z-value | p-value |
| --- | --- | --- | --- | --- |
| (Intercept) | -1.146e+02 | 8.637e+01 | -1.327 | 0.185 |
| GroupPlant | -2.193e-01 | 3.872e-01 | -0.566 | 0.571 |
| Start | -4.601e-03 | 3.449e-03 | -1.334 | 0.182 |
| End | 6.162e-02 | 4.318e-02 | 1.427 | 0.154 |
| Duration | NA | NA | NA | NA |

Appendix S2, Table 22. Results of GLM analyses comparing the frequency of local extinction in tropical animal and plant species on terrestrial, elevational gradients (504 species total).

|  | Estimate | Standard error | z-value | p-value |
| --- | --- | --- | --- | --- |
| (Intercept) | 0.0975 | 0.1072 | 0.910 | 0.363 |
| GroupPlant | 0.2812 | 0.1955 | 1.438 | 0.150 |

Appendix S2, Table 23. Results of GLM analyses comparing the frequency of local extinction in different groups of animals (716 species total), including study start date, end date, and duration.

|  | Estimate | Standard error | z-value | p-value |
| --- | --- | --- | --- | --- |
| (Intercept) | -85.85933 | 34.48469 | -2.490 | 0.0128 |
| **Taxonomic.groupAnnelida** | **1.32874** | **0.60780** | **2.186** | **0.0288** |
| Taxonomic.groupBird | 0.97113 | 0.54905 | 1.769 | 0.0769 |
| Taxonomic.groupCrustacea | 0.59737 | 0.70453 | 0.848 | 0.3965 |
| Taxonomic.groupEchinodermata | -0.36771 | 1.24974 | -0.294 | 0.7686 |
| **Taxonomic.groupFish** | **1.08861** | **0.55174** | **1.973** | **0.0485** |
| **Taxonomic.groupInsect** | **1.04391** | **0.52563** | **1.986** | **0.0470** |
| Taxonomic.groupMammal | 1.07674 | 0.70132 | 1.535 | 0.1247 |
| Taxonomic.groupMollusca | 0.79589 | 0.66256 | 1.201 | 0.2297 |
| Taxonomic.groupSquamate | 0.13110 | 0.77047 | 0.170 | 0.8649 |
| Start | -0.01899 | 0.05151 | -0.369 | 0.7123 |
| End | 0.06167 | 0.05400 | 1.142 | 0.2535 |
| Duration | -0.03496 | 0.05175 | -0.676 | 0.4993 |

Appendix S2, Table 24. Results of GLM analyses comparing the frequency of local extinction in different groups of animals (716 species total), including habitat, climatic region, and survey type (latitude or elevation).

|  | Estimate | Standard error | z-value | p-value |
| --- | --- | --- | --- | --- |
| (Intercept) | -0.91293 | 0.51636 | -1.768 | 0.077057 |
| Taxonomic.groupAnnelida | 2.67218 | 0.82557 | 3.237 | 0.001209 |
| Taxonomic.groupBird | 0.36089 | 0.50460 | 0.715 | 0.474484 |
| **Taxonomic.groupCrustacea** | **1.94081** | **0.89917** | **2.158** | **0.030894** |
| Taxonomic.groupEchinodermata | 0.97573 | 1.36894 | 0.713 | 0.475992 |
| Taxonomic.groupFish | 1.96898 | 0.65962 | 2.985 | 0.002836 |
| Taxonomic.groupInsect | 0.85936 | 0.49330 | 1.742 | 0.081499 |
| Taxonomic.groupMammal | -0.03264 | 0.62375 | -0.052 | 0.958271 |
| **Taxonomic.groupMollusca** | **1.89202** | **0.85088** | **2.224** | **0.026174** |
| Taxonomic.groupSquamate | 0.23256 | 0.75506 | 0.308 | 0.758086 |
| **HabitatMarine** | **-2.64300** | **0.67965** | **-3.889** | **0.000101** |
| HabitatTerrestrial | NA | NA | NA | NA |
| Climatic.regionTropical | 0.37393 | 0.20108 | 1.860 | 0.062937 |
| **Latitude.or.elevationLatitude** | **1.48159** | **0.43347** | **3.418** | **0.000631** |

Appendix S2, Table 25. Results of GLM analyses comparing the frequency of local extinction in different groups of animals (716 species total), including habitat, climatic region, survey type, start date, end date, and duration.

|  | Estimate | Standard error | z-value | p-value |
| --- | --- | --- | --- | --- |
| (Intercept) | -167.37624 | 50.70643 | -3.301 | 0.000964 |
| **Taxonomic.groupAnnelida** | **2.22491** | **0.83491** | **2.665** | **0.007702** |
| Taxonomic.groupBird | 0.07546 | 0.63432 | 0.119 | 0.905306 |
| Taxonomic.groupCrustacea | 1.49354 | 0.90776 | 1.645 | 0.099906 |
| Taxonomic.groupEchinodermata | 0.52846 | 1.37459 | 0.384 | 0.700644 |
| Taxonomic.groupFish | 1.04685 | 0.73417 | 1.426 | 0.153899 |
| Taxonomic.groupInsect | 0.42383 | 0.57439 | 0.738 | 0.460582 |
| Taxonomic.groupMammal | -0.52708 | 0.90313 | -0.584 | 0.559479 |
| Taxonomic.groupMollusca | 1.19871 | 0.89828 | 1.334 | 0.182055 |
| Taxonomic.groupSquamate | 0.10068 | 0.77241 | 0.130 | 0.896290 |
| **HabitatMarine** | **-2.63201** | **0.77830** | **-3.382** | **0.000720** |
| HabitatTerrestrial | NA | NA | NA | NA |
| Climatic.regionTropical | -0.31705 | 0.28811 | -1.100 | 0.271136 |
| **Latitude.or.elevationLatitude** | **1.48495** | **0.51300** | **2.895** | **0.003796** |
| Start | -0.01477 | 0.05316 | -0.278 | 0.781140 |
| End | 0.09823 | 0.05746 | 1.710 | 0.087358 |
| Duration | -0.01669 | 0.05373 | -0.311 | 0.756016 |
