# Supplementary material for: Climate-Related Local Extinctions Are Already Widespread among Plant and Animal Species
Source: PLoS Biol. 2016 Dec 8;14(12):e2001104. doi: 10.1371/journal.pbio.2001104 (PMC5147797; doi:10.1371/journal.pbio.2001104)
Supplement: S3 Appendix — (DOC) [file pbio.2001104.s003.doc]

Appendix S3. Results of GLMM analyses, showing variable coefficients.

Appendix S3, Table 1. Results of GLMM analyses including all 976 species, testing the effects of habitat (freshwater, marine, terrestrial), climatic region (tropical, temperate), the start date, end date, and duration of the study, taxonomic group (plants vs. animals), and whether the species were surveyed on a latitudinal or elevational gradient, on the frequency of local extinction

|  | Estimate | Standard error | z-value | p-value |
| --- | --- | --- | --- | --- |
| (Intercept) | -76.78159 | 49.93263 | -1.538 | 0.124121 |
| **HabitatMarine** | **-2.67899** | **0.73240** | **-3.658** | **0.000254** |
| **HabitatTerrestrial** | **-1.77474** | **0.53931** | **-3.291** | **0.000999** |
| **Climatic.regionTropical** | **0.89783** | **0.26548** | **3.382** | **0.000720** |
| Start | -0.02631 | 0.05152 | -0.511 | 0.609502 |
| End | 0.06509 | 0.05663 | 1.149 | 0.250380 |
| Duration | -0.02390 | 0.05145 | -0.465 | 0.642257 |
| GroupPlant | -0.65369 | 0.37505 | -1.743 | 0.081340 |
| **Latitude.or.elevationLatitude** | **1.76742** | **0.48145** | **3.671** | **0.000242** |

Appendix S3, Table 2. Results of GLMM analyses including all 976 species, testing effects of habitat, climatic region, taxonomic group (plants vs. animals), and whether the species were surveyed on a latitudinal or elevational gradient, on the frequency of local extinction.

|  | Estimate | Standard error | z-value | p-value |
| --- | --- | --- | --- | --- |
| (Intercept) | 1.0868 | 0.8176 | 1.329 | 0.1838 |
| **HabitatMarine** | **-2.7446** | **1.0944** | **-2.508** | **0.0121** |
| HabitatTerrestrial | -1.6160 | 0.8730 | -1.851 | 0.0642 |
| Climatic.regionTropical | 0.7114 | 0.4327 | 1.644 | 0.1001 |
| GroupPlant | -0.4669 | 0.4974 | -0.939 | 0.3478 |
| **Latitude.or.elevationLatitude** | **1.4872** | **0.5849** | **2.543** | **0.0110** |

Appendix S3, Table 3. Results of GLMM analyses including all 976 species, testing effects of habitat, climatic region, taxonomic group (plants vs. animals), survey type (latitudinal or elevational), and different geographic regions on the frequency of local extinctions.

|  | Estimate | Standard error | z-value | p-value |
| --- | --- | --- | --- | --- |
| (Intercept) | 0.69338 | 1.03298 | 0.671 | 0.50207 |
| **HabitatMarine** | **-2.43194** | **0.88869** | **-2.736** | **0.00621** |
| HabitatTerrestrial | -1.32471 | 0.71095 | -1.863 | 0.06242 |
| Climatic.regionTropical | 1.43445 | 0.74282 | 1.931 | 0.05347 |
| **GroupPlant** | **-0.97720** | **0.46695** | **-2.093** | **0.03637** |
| **Latitude.or.elevationLatitude** | **1.45391** | **0.53575** | **2.714** | **0.00665** |
| Geographic.regionEurope | 0.38629 | 0.81923 | 0.472 | 0.63726 |
| Geographic.regionMadagascar | -1.36147 | 0.74051 | -1.839 | 0.06598 |
| Geographic.regionNorth America | -0.05933 | 0.69292 | -0.086 | 0.93177 |
| Geographic.regionOceania (Hawaii) | 0.17411 | 1.18765 | 0.147 | 0.88345 |
| Geographic.regionOceania (New Guinea) | 0.26299 | 0.70802 | 0.371 | 0.71031 |
| **Geographic.regionSouth America** | **-1.70745** | **0.70208** | **-2.432** | **0.01502** |

**Appendix S3, Table 4. Results of GLMM analyses including all 976 species, testing effects of habitat, climatic region, taxonomic group (plants vs. animals), survey type (latitudinal or elevational), different geographic regions, and the start date, end date, and duration of the study, on the frequency of local extinction.**

|  | Estimate | Standard error | z-value | p-value |
| --- | --- | --- | --- | --- |
| (Intercept) | -75.36862 | 58.61793 | -1.286 | 0.198527 |
| **HabitatMarine** | **-2.59631** | **0.73912** | **-3.513** | **0.000444** |
| **HabitatTerrestrial** | **-1.65109** | **0.55729** | **-2.963** | **0.003050** |
| Climatic.regionTropical | 1.21344 | 0.84480 | 1.436 | 0.150899 |
| GroupPlant | -0.76520 | 0.65433 | -1.169 | 0.242225 |
| **Latitude.or.elevationLatitude** | **1.71062** | **0.50686** | **3.375** | **0.000738** |
| Geographic.regionEurope | 0.16490 | 0.98875 | 0.167 | 0.867544 |
| Geographic.regionMadagascar | -0.87366 | 0.60604 | -1.442 | 0.149416 |
| Geographic.regionNorth America | 0.10427 | 0.74804 | 0.139 | 0.889145 |
| Geographic.regionOceania (Hawaii) | 0.20908 | 1.24250 | 0.168 | 0.866366 |
| Geographic.regionOceania (New Guinea) | 0.33489 | 0.51821 | 0.646 | 0.518123 |
| **Geographic.regionSouth America** | **-1.52248** | **0.49603** | **-3.069** | **0.002145** |
| Start | -0.02496 | 0.05194 | -0.481 | 0.630786 |
| End | 0.06299 | 0.05832 | 1.080 | 0.280133 |
| Duration | -0.02374 | 0.05144 | -0.461 | 0.644499 |

Appendix S3, Table 5. Results of GLMM analyses including only terrestrial species surveyed on elevational gradients (805 species), testing effects of climatic region, the start date, end date, and duration of the study, and taxonomic group (plants vs. animals) on the frequency of local extinction.

|  | Estimate | Standard error | z-value | p-value |
| --- | --- | --- | --- | --- |
| (Intercept) | -104.38750 | 57.46070 | -1.817 | 0.06927 |
| **Climatic.regionTropical** | **0.83631** | **0.26713** | **3.131** | **0.00174** |
| **GroupPlant** | **-1.00764** | **0.40062** | **-2.515** | **0.01190** |
| Start | -0.02854 | 0.05151 | -0.554 | 0.57958 |
| End | 0.08015 | 0.05845 | 1.371 | 0.17025 |
| Duration | -0.02233 | 0.05145 | -0.434 | 0.66429 |

Appendix S3, Table 6. Results of GLMM analyses including only terrestrial species surveyed on elevational gradients (805 species), testing effects of climatic region and taxonomic group (plants vs. animals) on the frequency of local extinction.

|  | Estimate | Standard error | z-value | p-value |
| --- | --- | --- | --- | --- |
| (Intercept) | -0.4224 | 0.3106 | -1.360 | 0.174 |
| Climatic.regionTropical | 0.6191 | 0.4356 | 1.421 | 0.155 |
| GroupPlant | -0.5085 | 0.4988 | -1.020 | 0.308 |

Appendix S3, Table 7. Results of GLMM analyses including only plant species (260 species), testing effects of climatic region on the frequency of local extinction.

|  | Estimate | Standard error | z-value | p-value |
| --- | --- | --- | --- | --- |
| (Intercept) | -2.3671 | 0.3486 | -6.790 | 1.12e-11 |
| **Climatic.regionTropical** | **2.7457** | **0.3851** | **7.131** | **9.98e-13** |

Appendix S3, Table 8. Results of GLMM analyses including only terrestrial animal species surveyed on elevational gradients (545 species), testing effects of climatic region, the start date, end date, and duration of the study on the frequency of local extinction.

|  | Estimate | Standard error | z-value | p-value |
| --- | --- | --- | --- | --- |
| (Intercept) | -165.86837 | 62.65066 | -2.648 | 0.00811 |
| Climatic.regionTropical | -0.11358 | 0.33171 | -0.342 | 0.73205 |
| Start | -0.01233 | 0.05143 | -0.240 | 0.81056 |
| End | 0.09503 | 0.06009 | 1.581 | 0.11377 |
| Duration | -0.01419 | 0.05123 | -0.277 | 0.78178 |

Appendix S3, Table 9. Results of GLMM analyses including only terrestrial animal species surveyed on elevational gradients (545 species), testing effects of climatic region on the frequency of local extinction.

|  | Estimate | Standard error | z-value | p-value |
| --- | --- | --- | --- | --- |
| (Intercept) | -0.18361 | 0.29614 | -0.620 | 0.535 |
| Climatic.regionTropical | 0.07273 | 0.44173 | 0.165 | 0.869 |

Appendix S3, Table 10. Results of GLMM analyses including only bird species (233 species), testing effects of climatic region on the frequency of local extinction. Note that this analysis will not run if study dates (start, end, duration) are included.

|  | Estimate | Standard error | z-value | p-value |
| --- | --- | --- | --- | --- |
| (Intercept) | 0.06655 | 0.68273 | 0.097 | 0.922 |
| Climatic.regionTropical | 0.01083 | 0.98645 | 0.011 | 0.991 |

Appendix S3, Table 11. Results of GLMM analyses including only bird species (233 species), testing effects of climatic region, and the type of survey (elevational vs. latitudinal) on the frequency of local extinction. Note that this analysis will not run if study dates (start, end, duration) are included.

|  | Estimate | Standard error | z-value | p-value |
| --- | --- | --- | --- | --- |
| (Intercept) | -0.7963 | 0.5577 | -1.428 | 0.15334 |
| Climatic.regionTropical | 0.8707 | 0.7620 | 1.143 | 0.25318 |
| **Latitude.or.elevationLatitude** | **2.8459** | **0.9133** | **3.116** | **0.00183** |

Appendix S3, Table 12. Results of GLMM analyses including only insect species (271 species), testing effects of climatic region, the start date, end date, and duration of the study, and the type of survey (elevational vs. latitudinal) on the frequency of local extinction.

|  | Estimate | Standard error | z-value | p-value |
| --- | --- | --- | --- | --- |
| (Intercept) | 452.3807 | 506.9830 | 0.892 | 0.372 |
| Climatic.regionTropical | -0.6357 | 1.2242 | -0.519 | 0.604 |
| Latitude.or.elevationLatitude | 13.6816 | 1035.1578 | 0.013 | 0.989 |
| Start | 0.1326 | 0.2734 | 0.485 | 0.628 |
| End | -0.3581 | 0.3866 | -0.926 | 0.354 |
| Duration | 0.1570 | 0.2810 | 0.559 | 0.576 |

Appendix S3, Table 13. Results of GLMM analyses including only insect species (271 species), testing effects of climatic regionand the type of survey (elevational vs. latitudinal) on the frequency of local extinction.

|  | Estimate | Standard error | z-value | p-value |
| --- | --- | --- | --- | --- |
| (Intercept) | 0.30748 | 0.26346 | 1.167 | 0.243 |
| Climatic.regionTropical | -0.09719 | 0.29778 | -0.326 | 0.744 |
| Latitude.or.elevationLatitude | 14.25819 | 1028.91747 | 0.014 | 0.989 |

Appendix S3, Table 14. Results of GLMM analyses including only fish species (69 species), testing effects of habitat (freshwater vs. marine) on the frequency of local extinction. Note that these analyses will not run if study dates (beginning, end, duration) or survey type (latitude vs. elevation).

|  | Estimate | Standard error | z-value | p-value |
| --- | --- | --- | --- | --- |
| (Intercept) | 1.0561 | 0.4105 | 2.573 | 0.0101 |
| **HabitatMarine** | **-1.1615** | **0.5235** | **-2.219** | **0.0265** |

Appendix S3, Table 15. Results of GLMM analyses including only temperate animal species (367 species), testing effects of habitat (terrestrial, freshwater vs. marine), the start date, end date, and duration of the study on the frequency of local extinction.

|  | Estimate | Standard error | z-value | p-value |
| --- | --- | --- | --- | --- |
| (Intercept) | -95.413881 | 65.139661 | -1.465 | 0.143 |
| HabitatMarine | -0.754342 | 0.625317 | -1.206 | 0.228 |
| HabitatTerrestrial | -0.484924 | 0.617313 | -0.786 | 0.432 |
| Start | 0.000424 | 0.053340 | 0.008 | 0.994 |
| End | 0.047839 | 0.061945 | 0.772 | 0.440 |
| Duration | -0.016821 | 0.053164 | -0.316 | 0.752 |

Appendix S3, Table 16. Results of GLMM analyses including only temperate animal species (367 species), testing effects of habitat (terrestrial, freshwater vs. marine) on the frequency of local extinction.

|  | Estimate | Standard error | z-value | p-value |
| --- | --- | --- | --- | --- |
| (Intercept) | 1.0822 | 0.6813 | 1.588 | 0.112 |
| HabitatMarine | -1.2123 | 0.7714 | -1.572 | 0.116 |
| HabitatTerrestrial | -1.1278 | 0.7267 | -1.552 | 0.121 |

Appendix S3, Table 17. Results of GLMM analyses including only temperate animal species (367 species), testing effects of habitat (terrestrial, freshwater vs. marine) and type of survey (elevational vs. latitudinal) on the frequency of local extinction.

|  | Estimate | Standard error | z-value | p-value |
| --- | --- | --- | --- | --- |
| (Intercept) | 1.0821 | 0.6785 | 1.595 | 0.1108 |
| **HabitatMarine** | **-2.4195** | **0.9401** | **-2.574** | **0.0101** |
| HabitatTerrestrial | -1.3671 | 0.7314 | -1.869 | 0.0616 |
| **Latitude.or.elevationLatitude** | **1.2082** | **0.5419** | **2.230** | **0.0258** |

Appendix S3, Table 18. Results of GLMM analyses including only temperate animal species (367 species), testing effects of habitat (terrestrial, freshwater vs. marine), survey type, and the start date, end date, and duration of the study on the frequency of local extinction.

|  | Estimate | Standard error | z-value | p-value |
| --- | --- | --- | --- | --- |
| (Intercept) | -1.080e+02 | 6.601e+01 | -1.636 | 0.1019 |
| **HabitatMarine** | **-1.750e+00** | **8.098e-01** | **-2.160** | **0.0307** |
| HabitatTerrestrial | -6.792e-01 | 6.227e-01 | -1.091 | 0.2753 |
| Latitude.or.elevationLatitude | 9.976e-01 | 5.187e-01 | 1.923 | 0.0544 |
| Start | -3.027e-03 | 5.234e-02 | -0.058 | 0.9539 |
| End | 5.750e-02 | 6.144e-02 | 0.936 | 0.3493 |
| Duration | -1.743e-02 | 5.213e-02 | -0.334 | 0.7381 |

Appendix S3, Table 19. Results of GLMM analyses comparing the frequency of local extinction in temperate animal and plant species on terrestrial, elevational gradients (301 species total), including study start date, end date, and duration.

|  | Estimate | Standard error | z-value | p-value |
| --- | --- | --- | --- | --- |
| (Intercept) | -1.434e+02 | 8.914e+01 | -1.609 | 0.1076 |
| **Taxonomic group** | **-2.130e+00** | **8.442e-01** | **-2.523** | **0.0116** |
| Start | -8.904e-03 | 5.228e-02 | -0.170 | 0.8648 |
| End | 8.057e-02 | 6.956e-02 | 1.158 | 0.2467 |
| Duration | -1.542e-02 | 5.205e-02 | -0.296 | 0.7671 |

Appendix S3, Table 20. Results of GLMM analyses comparing the frequency of local extinction in temperate animal and plant species (301 species total) on terrestrial, elevational gradients.

|  | Estimate | Standard error | z-value | p-value |
| --- | --- | --- | --- | --- |
| (Intercept) | -0.2281 | 0.2494 | -0.914 | 0.360475 |
| **Taxonomic group** | **-2.1711** | **0.6258** | **-3.469** | **0.000522** |

Appendix S3, Table 21. Results of GLMM analyses comparing the frequency of local extinction in tropical animal and plant species on terrestrial, elevational gradients (504 species total). Note that this analysis will not run if study dates (start, end, duration) are included.

|  | Estimate | Standard error | z-value | p-value |
| --- | --- | --- | --- | --- |
| (Intercept) | -0.08933 | 0.29303 | -0.305 | 0.760 |
| Taxonomic group | 0.39273 | 0.49508 | 0.793 | 0.428 |

Appendix S3, Table 22. Results of GLMM analyses comparing the frequency of local extinction in different groups of animals (716 species total), including study start date, end date, and duration.

|  | Estimate | Standard error | z-value | p-value |
| --- | --- | --- | --- | --- |
| (Intercept) | -85.85933 | 47.74846 | -1.798 | 0.0722 |
| Taxonomic.groupAnnelida | 1.32874 | 0.73459 | 1.809 | 0.0705 |
| Taxonomic.groupBird | 0.97113 | 0.65956 | 1.472 | 0.1409 |
| Taxonomic.groupCrustacea | 0.59737 | 0.81630 | 0.732 | 0.4643 |
| Taxonomic.groupEchinodermata | -0.36771 | 1.31501 | -0.280 | 0.7798 |
| Taxonomic.groupFish | 1.08861 | 0.65703 | 1.657 | 0.0975 |
| Taxonomic.groupInsect | 1.04391 | 0.63402 | 1.647 | 0.0997 |
| Taxonomic.groupMammal | 1.07674 | 0.84255 | 1.278 | 0.2013 |
| Taxonomic.groupMollusca | 0.79589 | 0.77308 | 1.030 | 0.3032 |
| Taxonomic.groupSquamate | 0.13110 | 0.77322 | 0.170 | 0.8654 |
| Start | -0.01900 | 0.05236 | -0.363 | 0.7167 |
| End | 0.06167 | 0.05684 | 1.085 | 0.2779 |
| Duration | -0.03496 | 0.05266 | -0.664 | 0.5068 |

Appendix S3, Table 23. Results of GLMM analyses comparing the frequency of local extinction in different groups of animals (716 species total).

|  | Estimate | Standard error | z-value | p-value |
| --- | --- | --- | --- | --- |
| (Intercept) | -0.38383 | 0.73195 | -0.524 | 0.600 |
| Taxonomic.groupAnnelida | 0.76365 | 0.96776 | 0.789 | 0.430 |
| Taxonomic.groupBird | 0.36747 | 0.79891 | 0.460 | 0.646 |
| Taxonomic.groupCrustacea | 0.02241 | 1.03118 | 0.022 | 0.983 |
| Taxonomic.groupEchinodermata | -0.95453 | 1.46264 | -0.653 | 0.514 |
| Taxonomic.groupFish | 0.68319 | 0.84612 | 0.807 | 0.419 |
| Taxonomic.groupInsect | 0.65208 | 0.80866 | 0.806 | 0.420 |
| Taxonomic.groupMammal | -0.35531 | 0.88867 | -0.400 | 0.689 |
| Taxonomic.groupMollusca | 0.10833 | 0.94912 | 0.114 | 0.909 |
| Taxonomic.groupSquamate | 0.17836 | 0.75787 | 0.235 | 0.814 |
